# A critical incident study of ICU nurses during the COVID-19 pandemic

**DOI:** 10.1177/09697330211043270

**Published:** 2021-12-05

**Authors:** Ann Rhéaume, Myriam Breau, Stéphanie Boudreau

**Affiliations:** Université de Moncton, Canada

**Keywords:** COVID-19, critical incident technique, intensive care nurse, pandemic, qualitative study

## Abstract

**Background:**

Intensive care unit nurses are providing care to COVID-19 patients in a stressful environment. Understanding intensive care unit nurses’ sources of distress is important when planning interventions to support them.

**Purpose:**

To describe Canadian intensive care unit nurse experiences providing care to COVID-19 patients during the second wave of the pandemic.

**Design:**

Qualitative descriptive component within a larger mixed-methods study.

**Participants and research context:**

Participants were invited to write down their experiences of a critical incident, which distressed them when providing nursing care. Thematic analysis was used to analyze the data.

**Ethical considerations:**

The study was approved by the ethics committee at the researchers’ university in eastern Canada.

**Results:**

A total of 111 critical incidents were written by 108 nurses. Four themes were found: (1) managing the pandemic, (2) witness to families’ grief, (3) our safety, and (4) futility of care. Many nurses’ stories also focused on the organizational preparedness of their institutions and concerns over their own safety.

**Discussion:**

Nurses experienced moral distress in relation to family and patient issues. Situations related to insufficient institutional support, patient, and family traumas, as well as safety issues have left nurses deeply distressed.

**Conclusion:**

Identifying situations that distress intensive care unit nurses can lead to targeted interventions mitigating their negative consequences by providing a safe work environment and improving nurses’ well-being.

## Introduction

According to the World Health Organization (WHO), protection and care of frontline workers during the pandemic is a priority as they provide essential care.^1^ Healthcare workers have adapted to a quickly changing environment, providing complex care to patients with new guidelines being implemented frequently.^2,3^ They are at risk for several psychological problems, such as anxiety, depression, and post-traumatic stress disorder (PTSD) symptoms as a result of the stressful work environment associated with providing care to infected patients.^4^ Moreover, frontline healthcare workers have a three-fold increased risk of infection compared to the general community because of their exposure to COVID-19 patients.^5^ The International Council of Nurses (ICN) estimates that more than 1500 nurses have died of COVID-19 as of October 2020, an increase of 67% from 2 months earlier.^6^ This is believed to be grossly underestimated. Although there is no published data on the number of nurse deaths in Canada, as of 15 January 2021, 65,920 Canadian healthcare workers have been infected with COVID-19 and 24 of these have died.^7^

Although there is an increasing number of qualitative studies describing the experiences of nurses during the pandemic,^2,8^ there are few empirical studies exploring ICU nurse experiences. Existing studies on ICU nurses highlight many problematic issues during the pandemic, such as excessive workloads,^3,9,10^ rapidly changing and unclear information,^9^ shortage of personal protective equipment (PPE),^3,10^ and poor organizational support.^10^ Among different healthcare workers, ICU nurses are at an increased risk of infection as they spend more time directly caring for unstable COVID-19 patients requiring mechanical ventilation.^11^ A study examining psychological distress in ICU healthcare workers during COVID-19 showed that while 59% reported good well-being, 45% of participants met the threshold for one of the following disorders: depression, PTSD symptoms, severe anxiety, and problem drinking.^12^ Moreover, ICU nurses in this study reported more mental health problems than all other staff and one in five nurses reported thoughts of self-harm or suicide.

Nurses are confronting many ethical challenges during the pandemic^8,13,14^ and ICU nurses, in particular, are at a greater risk of experiencing patient-related issues causing moral distress.^15^ Furthermore, ICU nurses have the highest prevalence rates of burnout among different specialties, which is often related to moral distress as well as other unique characteristics of the critical care environment (e.g. complex patient care).^16^ This being the case, the pandemic may further amplify the stressful work environment of these nurses. Given the paucity of studies exploring recent ICU nurse experiences, identifying sources of distress are important to identify both unit-level and hospital-level interventions to mitigate the effects of the pandemic.

### Purpose

The purpose of this study is to explore the causes of distress in ICU nurses during the pandemic.

## Methods

### Design

This study is part of a larger concurrent mixed-methods study examining the impact of COVID-19 on nurses working in ICUs in Canada. The project consisted of two phases: (1) a cross-sectional component and (2) a written qualitative component. This article focuses on the qualitative component.

### Setting and sample

A convenience sample of ICU nurses was recruited from the Canadian Association of Critical Care Nurses (CACCN). The CACCN sent an initial email request followed by two reminders at 3-week intervals inviting members to respond to an online questionnaire. Social media, such as Facebook, were also used to distribute the survey through organizations affiliated with the CACCN. A total of 1400 ICU nurses were asked to participate and, of these, 236 responded to the online survey and 108 responded to the qualitative component of the survey.

### Data collection

**Figure 1.**
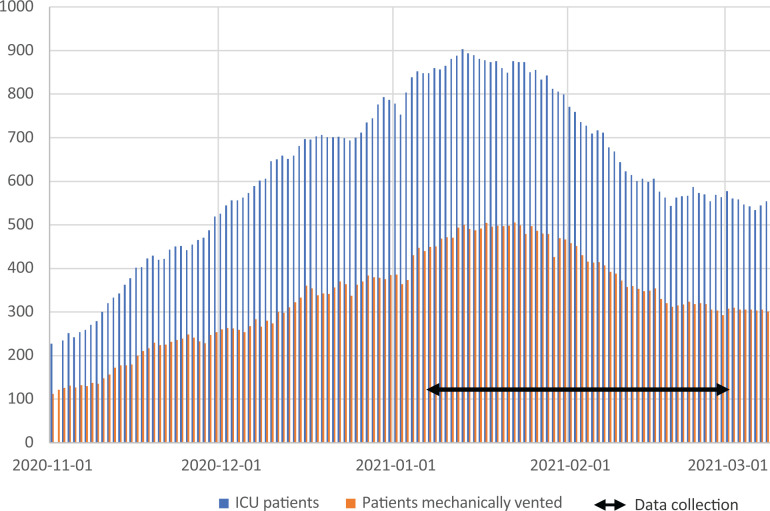
Number of ICU patients pre and post data collection.

This study took place during the second wave of the COVID-19 pandemic in Canada. Data were collected from 11 January to 2 March 2021 (Figure 1). The first COVID-19 case was reported on 25 January 2020 in Canada and the daily number of cases peaked at 2560 on 3 May 2020 during the first wave.^7^ The second wave began in September 2020, with a peak of 6549 cases on 9 November 2020.

We used the critical incident technique (CIT) developed by Flannagan.^17^ CIT focuses on the description of actual events occurring in the past. These events may involve behaviors or activities affecting certain outcomes and are meaningful to those involved.^18^ Working retrospectively, the person is asked to reflect on behaviors or actions that could have led to an outcome or result. Thus, researchers attempt to gain an understanding of participant experiences and the behaviors or actions taken to handle the situations.^18,19^ Over the years, CIT has been increasingly used in healthcare research and nursing, in particular.^19,20^ The guide on CIT by Schluter et al.^18^ was used for the development of questions and ensuing analysis as it focused on CIT’s applicability in nursing research and outlines how the basic CIT questions can be modified for issues relevant to nurses. Participants were initially asked to describe a situation when caring for a COVID-19 patient made them feel distressed. The situation could refer directly to patient care, equipment used, or any other aspect within the ICU setting. Following this, five questions were asked regarding the particular situation (see Table 1).

**Table 1.** CIT questions.

Can you describe an incident or a situation when caring for a COVID-19 patient that made you feel distressed? It can be related to any aspect (patient care, equipment, etc.).
1. Reflect upon the incident: what made it critical from your point of view?
2. What preceded and contributed to the incident?
3. What was the outcome or result?
4. What made this outcome effective or ineffective?
5. What could have made the outcomes more effective?

CIT: critical incident technique.

### Data analysis

The critical incidents were analyzed using thematic analysis according to Braun and Clarke^21^ with the assistance of Dedoose software.^22^ Data analysis occurred in two phases and used an inductive approach. Initially, each story was read several times to get a basic understanding of the event from the nurse’s perspective. For some participants, recollections of incidents were short, revealing little about the situation itself. Other participants had detailed descriptions of events and ensuing actions. During the second phase, initial codes were attributed to each document containing the critical incidents. We also coded all content in relation to the incidents, as they further explained participant thoughts and actions. Three separate members of the team created the initial codes. After several documents were coded, the team met regularly and discussed the coding system until consensus was reached. The codes were collapsed into categories mid-way through the analysis, and finally, broader themes. At the end, the team collectively confirmed the final themes. Data saturation was obtained halfway through the analysis; we nonetheless continued analysis with the existing codes since the data were already collected. A total of 111 critical incidents were identified and several participants shared more than one incident. The time completing the survey ranged from 20 min to 24 h as the survey allowed participants to save their responses and complete their answers later.

### Trustworthiness

The following criteria were used to establish trustworthiness of data. Credibility was enhanced through investigator triangulation as three members of the research team coded many passages separately and interpreted the data throughout the project. To promote transferability, we described the context of the pandemic during data collection in Canada and the characteristics of the participants. Confirmability was sought through an audit trail detailing decisions made by the research team from the beginning of the study throughout data analysis. The researchers maintained reflexivity as team members discussed how their own experiences could influence the analysis. One team member had over 10 years of experience as an ICU nurse.

### Ethical consideration

The study was approved by the ethics committee at the Université de Moncton on 9 December 2020 (approval number 2021-024). The survey included a cover letter guaranteeing the confidentiality and anonymity of the participants. Participants were informed of the study rationale in the cover letter of the survey. Consent was assumed when the participants completed the online questionnaire. The participants were also given a link to the *Canadian Psychological Association* with a list of psychologists in each province. The psychologists were available to provide telephone consultation to all frontline healthcare workers at no charge, in the event that participants felt distressed after responding to the survey.

## Results

The majority of participants were women (93%) and the average age was 36 years. Table 2 shows the socio-demographic data of the sample. Most participants were staff nurses (90.7%) and had an average of 11 years’ experience in ICU. In addition, 83% of the participants had full-time status. Although some participants worked in smaller hospitals, many worked either in medium-sized hospitals (38%) or large hospitals (50%).

**Table 2.** Socio-demographic characteristics of sample.

Characteristics	Frequency	Percentage
Age	Mean = 35.62	SD = 10.64
Experience in ICU	Mean = 10.61	SD = 9.70
Gender		
Female	99	93.4
Male	7	6.6
Title		
Staff nurse	97	90.7
Resource nurse	2	6.6
Other	10	1.4
Employment status		
Full-time	84	83.2
Part-time	11	10.9
Occasional	6	5.9
Hospital size		
Small (1–99 beds)	13	12.4
Medium (100–399 beds)	40	38.1
Large (400 beds and over)	52	49.5

SD: standard deviation; ICU: intensive care unit.

The following four themes emerged from the data: (1) managing the pandemic, (2) witness to families’ grief, (3) our safety, and (4) futility of care. Table 3 shows the themes derived from the critical incidents and subthemes emerging from the data.

**Table 3.** Themes, subcategories, and actions desired by participants.

Themes derived from critical incidents	Number of incidents	Subcategories	Actions desired by participants
Managing the pandemic	44	Constantly changing guidelines Limited resources and supplies Working short staffed	Clear expectations and guidelines, more education Increase PPE Ensuring protocols are followed Disciplinary action for those that break policies Increase staff numbers Involving staff in unit decisions
Witness to families’ grief	25	Dying alone Saying goodbye Trying to support families	Support for nurses following distressful events: – Team huddle – Counseling – Resources for nurses to talk about experiences
Our safety	24	Being exposed Physician decisions Administrative support	Policies to protect staff Better leadership Administrators listening to staff and being present
Futility of care	18	Prolonging hope Prolonging suffering	Consistent communication plan Open conversations about patient code status Increased provision of comfort care

PPE: personal protective equipment.

### Managing the pandemic

Nurses questioned the preparedness of their institutions. The concerns were based on three issues they believed were problematic: frequent policy and guideline changes, lack of resources and staffing shortages. Policies and guidelines changed frequently, sometimes day-to-day, which from nurses’ perspective was far too often. These rapid procedural changes left nurses feeling anxious and vulnerable. At some level, nurses understood that the rapid increase in knowledge of treatment modalities would be reflected in the care of critically ill patients. Nonetheless, nurses found this troublesome because they believed that many knowledge gaps arising during the first wave should have been resolved before the second wave occurred. Examples were given of policies regarding patient visits, isolation requirements, PPE, and patient code status.

The guidelines about which mask to use in which situation seems to be continually changing. With things constantly changing it makes it difficult to stay on top of things as well as you question the reasons behind some of the changes. (Participant 64)

Nurses also felt that constant changes in infection control guidelines were not always based on evidence. The consequences of this were a deep-rooted distrust of management.

Nurses spoke about the lack of resources within the ICUs. These resources were related to shortages of equipment, lack of human resources, and limited negative pressure rooms. Management dealt with regular PPE shortages in different ways, often resulting in a lack of transparency about PPE availability. Nurses were sometimes asked to reuse PPEs or make do with what they had. One nurse noted that her supervisor asked her to reuse isolation gowns as they were running short, while another supervisor asked the nurses to bring their isolation gowns home to wash. Faced with unsatisfactory actions, nurses went to the next level of management to acquire the appropriate protective gear. Nurses felt that they were at high risk of contagion and blamed management for not being prepared to handle the high number of COVID-19 cases during the second wave. One nurse noted: “We did not have a plan, nor coordination to manage high-intensity situations while maintaining these precautions. It takes discipline that comes from training, and we had no training” (participant 104). Nurses indicated they wanted clearer guidelines, more education, and involvement in unit decisions.

ICUs were frequently short staffed, which left nurses feeling exhausted. Staff from other areas were brought in and quickly trained to assist in caregiving, nonetheless, this also exacerbated the situation as deployed staff were not ICU nurses. One nurse explained: “Overwhelmed with ventilated COVID-19s, not enough staff working in a makeshift unit, plastic duct-taped walls with no end in sight” (participant 43). Makeshift units were quickly created to accommodate the increasing patients, but there were often issues with the implementation of these new spaces. As a result, nurses felt that the shortage of ICU nurses compromised patient care. Another nurse noted, “Understaffed intensive care unit is a perfect ground for mishaps and accidental deaths. Which happened to my hospital” (participant 14). Nurses believed that the shortage of ICU nurses and the increased numbers of unexpected COVID-19 ICU beds compromised patient care and safety. They asked for better management of personnel and beds.

### Witness to a family’s grief

Many critical incidents centered around the patient’s family. Most ICUs had a no-visitor policy with COVID-19 patients, which meant that the majority of patients died without their families by their side. Nurses were deeply disturbed by patients dying alone and had difficulty coming to terms with this situation. Similarly, families were distraught because their love ones were dying alone. One nurse stated “I should not be the last voice they (patients) hear. It should be someone they love” (participant 39). Nurses acted as an intermediary communicating between family and patients. This had its own set of challenges as patients were often intubated and not responding. IPads with Facetime were used and enabled family members to say their last goodbyes. The use of Facetime was very distressing for both families and nurses. Moreover, time constraints made it difficult dealing with many competing interventions. Some nurses resented having to prioritize iPad use (e.g. adjusting the angle of the iPad to give the family a view of the patient that would be the least upsetting for them) over providing complex care to patients who were proned with multiple lines.

The acuity of these situations was amplified when there were cultural or language barriers between nurses and the patient’s family. Examples were given of families who had recently come to Canada whereby a family member became ill with COVID-19 and was transferred to ICU. Interpreters were used to help with communication, nonetheless, the tragedy of becoming critically ill against a backdrop of being introduced to different customs in a new country was accentuated.

All along the interpreter was used to communicate with this poor wife who was already stressed and overwhelmed with all that was happening. Then with IPad in hand and pressed up to the glass window of the patients’ room, the family prayed, and then watched us take him off the ventilator…Also, while taking him off the vent (*ventilator*), the wife was shouting, “don’t take the organs” as though this was the only English she was able to make sure she got across to us. This was a gut wrenching, yet moment of absolute defeat. (Participant 13)

Nurses felt powerless to help these families and stated that they would often think of them afterward.

Nurses also described situations where the patients’ status would deteriorate rapidly, catching families unaware and unprepared. Furthermore, they had little time to prepare the family for the patient’s eventual death. Some nurses discussed the social worker’s role in supporting the families and felt that the team pulled together to help families through the ordeal. Other nurses were left alone to deal with family care and resented bearing the responsibility of communicating with the family with regards to the patient’s prognosis. In these cases, other healthcare workers took a back seat to family interventions because of fear of contagion. Nurses generally felt powerless to change the outcomes of care and guilt that they could not do more to alter patient outcomes. Several participants indicated that they have provided care to over 100 COVID-19 positive patients. One participant expressed,

But I have had 3 patients die suddenly, shortly after admission, despite our best efforts. And then when medical interventions fail and the family get the bad news, they don’t understand. And I know their grief and understanding is interrupted and I can’t fix that…And I feel guilty that the family has this trauma to process, and I couldn’t do more for them to help them emotionally heal. (Participant 69)

They appeared fatalistic about the outcomes, knowing that the odds were that the patients would not recover, or if they did, they would still have serious ramifications.

### Our safety

Over one-third of nurses’ stories touch directly or indirectly on concerns over their own and their colleagues’ safety. Most nurses believed that their safety was compromised when providing care and reported many incidents where they were exposed to COVID-19 at work. Faulty equipment at the bedside, such as aerosols, were often responsible for exposing nurses to the virus. One nurse stated “All my colleagues and I have been exposed for long periods to COVID-19 pts on aerosolized oxygen sources—didn’t even result in a good outcome for the patient” (participant 30). The broader physical environment also compromised nurse safety. Nurses described working in small rooms with full protective gear, making it difficult to move quickly when an incident occurred placing them at risk.

Nurses blamed a combination of poor physician decisions, management ineptitude, and lack of PPE supplies for situations, which increased their risk of contagion. Some physicians ignored public health guidelines in relation to testing patients for COVID-19. For instance, patients that were potentially a risk for COVID-19 were not always swabbed for testing, relying on physicians’ discretion instead of established protocols.

An MD (*medical doctor)* deciding to cancel a pending COVID-19 swab because she didn’t “feel” like they had COVID-19. And then later on that patient being swabbed and coming back positive—meaning that staff who are not allowed to work with COVID-19 patients (for medical exemption reasons) have been put at risk. (Participant 42)

Patients who were initially classified as non-COVID-19 risks were then reclassified as a COVID-19 risk days later. This led to a short period of time whereby nurses did not have full PPE when caring for these patients who were contagious all along. Furthermore, some of these patients, prior to being tested positive for COVID-19, were placed in open units near the nursing station where nurses did not consistently wear protective gear. The shortage of PPEs and ensuing rationing of equipment also put nurses at risk of infection. A nurse stated: “Last year when N95 supply was low, last year when surgeries continued; patient in our ICU open bay was COVID-19 positive and intubated—this infected 6 staff members” (participant 28).

Nurses wanted more input into decisions regarding patient care and unit policies. Participants considered this a right because they were putting themselves at risk caring for contagious patients in a less than optimal environment. Nonetheless, numerous examples were given of both physicians and managers making decisions without consulting nurses. These situations angered nurses as they discussed the ramifications of these decisions in relation to their own safety. Nurses’ stories highlighted communication between management and nurses, which appeared to be top-down. One nurse noted that she wished management would respect them more, stating: “Respecting what makes an employee feel safe, especially when it is sound and justified, and not hold safety measures and equipment hostage from them” (participant 66). Others felt that managers would not be making the same decisions if they had to work on the front line. The outcome left frontline ICU nurses feeling alone.

### Futility of care

Many nurses expressed distress about providing care that they considered would not benefit the patient. At the heart of the issue was giving complex, painful treatments to patients who had questionable benefits. Nurses believed that patients would be better served if they were provided palliative care. Nurses predominantly blamed families for wanting to prolong the life of their loved ones, although they held physicians accountable for letting themselves be pressured by families and unwilling to make decisions in the patient’s best interests. In some cases, nurses noted that physicians would equivocate when faced with difficult situations. Participants believed that physicians lacked the moral courage to make difficult decisions that would make it clear to families that the patient would not be going home. Nurses were not involved in the decision-making process in relation to appropriate treatments at the end of life.

The outcome was a double-edge sword. Continuing treatments with questionable benefits were often painful yet, in the short term, provided traumatized families with hope that the patient could survive. Nonetheless, by prolonging false hope, the medical team also prolonged patient suffering. At the end, nurses noted that most patients died. One nurse stated what was most stressful for her was the following: “…constant ethical dilemma of keeping someone alive when there is no hope for recovery and keeping patient alive at the cost of the patient’s comfort, i.e. constantly inflicting painful procedures” (participant 52). Some families demanded treatments, which were based on their personal or religious beliefs, going against the patients’ own wishes who were no longer able to communicate.

Nurses identified the changes that they would like to see. Participants wanted more open discussions with families and all team members to realistically examine the patients’ prognosis. They believed that this did not occur often during the pandemic. One nurse described her patients in the following way:

All patients over 80 years of age, with multiple comorbidities and on a ventilator with COVID-19. Family members and doctors want to keep everything going even though patient has been proned and intubated, paralyzed and sedated for over 2 weeks, with little chance of recovering with any kind of quality of life. (Participant 32)

Moreover, many nurses indicated that they wanted more consistency in terms of following the initial plan of care and being less influenced by families, who are often in a state of crisis. The physicians’ indecision regarding care treatment would often lead to increased suffering of patients who were not able to speak for themselves. Nurses believed that meetings with families needed to be more transparent in terms of discussion of the patient’s prognosis, which could inform decisions and alleviate patient suffering.

## Discussion

This study explored ICU nurses’ experiences when providing care to COVID-19 patients through critical incident narratives. Our findings show a brief snapshot of ICU nurses’ work environments during the pandemic and are consistent with studies in a range of countries with a continuum of different healthcare systems.^3,9,23^ Many critical incidents centered around how the pandemic was managed. The lack of clear guidelines, rationing of PPEs, and shortage of personnel led ICU nurses to question the preparedness of their institutions in managing the outbreak and contributed to their distress. Organizational preparedness is one of the major factors in determining how frontline nurses deal with the pandemic,^2^ while insufficient organizational support is considered a major barrier in caring for COVID-19 patients.^24^ Policies and guidelines that are updated daily create confusion,^2^ anxiety, and distress.^3,25^ As LoGiudice and Bartos^26^ aptly point out, nurses are educated to rely on evidence-based knowledge. Quickly changing policies undermine this and suggest that decisions are being made on the fly, which may be deeply disconcerting for nurses.

Above all, nurses’ stories reflect a deep moral distress centered around two situations: patients dying alone and the inability to prevent patients from receiving lengthy painful treatments with questionable benefits. Several factors can lead to the development of moral distress in nurses, including specific clinical situations, internal constraints (e.g. powerlessness), and external constraints (e.g. communication with team members).^27,28^ Moral distress is prevalent in ICU nurses^27,29,30^ and mostly related to clinical situations, such as end-of-life issues.^15,31^ Furthermore, medical futility is the most common among the end-of-life issues.

Limited resources and high demands have brought decision-making regarding the appropriateness of futile treatment in seriously ill COVID-19 patients to the forefront.^32^ Patients are often not able to participate in end-of-life decisions and physicians themselves are less able to make effective decisions when confronted with families that require time processing the rapidly changing events occurring with their loved ones.^33^ Our participants did not play a prominent role in decisions related to treatment status. They understood that decisions to prolong treatment may not result in any quality of life and felt powerless to advocate for their patients. This is unfortunate, given that ICU nurses are well positioned to initiate end-of-life discussions with families^34^ and often prefer to be included in decisions regarding treatments.^35^ Nurses in our study experienced anger, powerlessness, and high levels of anxiety, characteristics reflecting moral distress in nurses.^31^ These situations have a detrimental effect on nurses’ well-being^27,36^ and may leave nurses with less time to care for other patients.^36^ The nature of the pandemic has exacerbated moral distress in ICU nurses as complex clinical situations arise when resources are scarce, system demands are exceedingly high and unpredictable.^15^ Working within these difficult circumstances, ICU nurses are less able to provide compassionate end-of-life care.

The sense of powerlessness was prevalent throughout nurses’ stories. Nurses wanted their voices heard in relation to patient care and unit management, yet their decision-making authority appeared to be severely limited within their settings. We believe that the pandemic has exacerbated the already existing difficult workplace environment of ICU nurses. Theorell^37^ argues that the pandemic magnifies the psychosocial risk factors for healthcare workers such as high demands, lack of control over work environment, and poor institutional support. These, in turn, create the underlying conditions for depression and burnout. Interestingly, nurses in some jurisdictions have acquired a larger scope of practice in relation to clinical and pharmaceutical management decisions as a result of the pandemic.^9^ Nonetheless, the shifting of certain competencies may not be the kind of autonomy that ICU nurses desire to successfully advocate for critically ill patients. Furthermore, a lack of autonomy is considered a main trigger in causing moral distress,^38^ as experienced by our participants.

Our participants had many concerns over their personal safety. They felt abandoned by management who were unwilling to respond to their basic needs. Concerns over personal safety and contagion have been expressed by many healthcare workers.^2,9,10^ Although the foundation of nursing is the duty to care, McKenna^39^ argues that “nurses must balance this duty of care for patients with their duty of care to themselves and their family members” (p. 1). Failure to do so could result in moral distress. Many nurses have felt insufficiently supported by their institutions during the pandemic.^9,14^ Moreover, organizational support is associated with reduced anxiety in nurses.^40^ Employers have a responsibility to provide a safe working environment for nurses and, according to the Canadian Nurses Association Code of ethics, both employers and governments have a duty to protect nurses during disasters and pandemics.^41^ This includes transparent communication, improving the state of preparedness of healthcare organizations, appropriate staffing levels and providing different types of support for nurses in relation to childcare and self-isolation.

The participants identified several interventions, which could be implemented at little cost that could mitigate some of the harmful effects of the pandemic, such as team huddles, management visibility, and a comprehensive communication plan. There are several excellent guidelines on support of healthcare workers during the pandemic that can be used as building blocks for interventions.^42,43^ Self-care strategies such as building moral resilience with targeted interventions (e.g. practicing mindfulness) can also help decrease moral distress.^42^ Healthcare organization leaders must be visible to answer questions and listen to staff concerns. They must also be open to discussing ethical issues confronting nurses. Supporting nurses through access to counseling, regular interdisciplinary meetings, and participation in unit management decisions will increase their sense of control of their work environment.

### Limitations

The use of CIT allowed us to describe situations that ICU nurses experience across the country. This is beneficial when dealing with a country as large as Canada. An advantage of using this methodology is that it enabled nurses to explore specific events occurring in their work environment. Using a web-based survey as a means to collect data also allowed nurses to express themselves freely when they were able to. It also gave participants the opportunity to write as little or as much as they wanted to. One of the main limitations of written CIT is that, unlike in-depth interviews, we were unable to probe further and elicit more information from participants. A second limitation was that our data gave us only one perspective of events unfolding within ICU settings. The perspective of physicians, frontline and middle managers would have greatly enriched our understanding of the complexities of the work environment during the pandemic.

## Conclusion

We have concluded the analysis of this study at the beginning of the third wave of the pandemic in Canada. Through descriptions of real events, this study contributes to a better understanding of the challenges facing ICU nurses as they provide care to critically ill patients. Nurses suffered from moral distress, worked in a chaotic unsafe environment with rapidly changing rules and felt abandoned by management. More can be done to improve this situation.

The pandemic has placed enormous pressure on healthcare workers such as ICU nurses. Failure to act may result in both short-term and long-term consequences on ICU nurses’ health and consequently the retention of a highly qualified workforce.
